# Association between weight loss and reproductive outcomes among women with overweight or obesity: a cohort study using UK real-world data

**DOI:** 10.1093/humrep/deaf122

**Published:** 2025-07-06

**Authors:** Maximiliane Lara Verfürden, Volker Schnecke, Eva Winning Lehmann, Adriana Rendón Guillén, Adam H Balen

**Affiliations:** Data Orchestration, Novo Nordisk A/S, Søborg, Denmark; Real World Evidence, Novo Nordisk A/S, Søborg, Denmark; Medical and Science, Obesity, Novo Nordisk A/S, Søborg, Denmark; Global Medical Affairs, Obesity, Novo Nordisk A/S, Søborg, Denmark; Leeds Centre for Reproductive Medicine, Leeds Teaching Hospitals, Leeds, UK

**Keywords:** body mass, fertility, gestational age, live birth, caesarean section, gestational diabetes, pregnancy-induced hypertension, real-world data

## Abstract

**STUDY QUESTION:**

In women with BMI ≥25 kg/m^2^, does a 10–25% weight loss versus stable weight increase the chance of pregnancy?

**SUMMARY ANSWER:**

In women with overweight or obesity, weight loss was associated with an increase in the chance of pregnancy.

**WHAT IS KNOWN ALREADY:**

Weight loss has been shown to improve conception rates among women with overweight or obesity and concomitant polycystic ovary syndrome (PCOS). However, evidence on the effect of weight loss on conception rates among the general population of women with overweight or obesity, irrespective of PCOS status, is lacking.

**STUDY DESIGN, SIZE, DURATION:**

A large cohort study of patient data collected from primary-care practices linked to hospital records in England between January 2000 and May 2022.

**PARTICIPANTS/MATERIALS, SETTING, METHODS:**

Women were included if they were aged 18–40 years with BMI ≥25 kg/m^2^. Patient data were extracted from the UK Clinical Practice Research Datalink Aurum database of electronic medical records. The primary outcome was the first pregnancy recorded during a 3-year follow-up period. Key secondary outcomes were the occurrence of miscarriage, gestational diabetes, and pregnancy-induced hypertension, as well as emergency caesarean section, and risk of babies being born large for gestational age (LGA).

**MAIN RESULTS AND THE ROLE OF CHANCE:**

The final cohort of 246 670 women comprised 195 666 who kept a stable weight and 51 004 who lost weight. Pregnancy occurred in 22 756/246 670 (9.2%) women. On average, a 10–25% weight loss (median 14%) was associated with a 5.2% increase in the chance of pregnancy over the following 3 years (hazard ratio 1.05; 95% CI 1.02, 1.09; *P *= 0.003) compared with stable weight. Gestational diabetes was reported for 950/11 825 (8.0%) women, and weight loss reduced the risk of gestational diabetes by 42% (odds ratio [OR] 0.58; 95% CI 0.48, 0.70; *P *< 0.001). Emergency caesarean section was performed in 1453/11 558 (12.6%) pregnancies. This intervention was significantly reduced in the weight-loss cohort (OR 0.82; 95% CI 0.71, 0.95; *P *= 0.008). Pregnancy-induced hypertension was reported in a few women (244/11 740 [2.1%]) and one-tenth of women (791/7988 [9.9%]) gave birth to babies who were LGA. Weight loss prior to pregnancy resulted in non-significant reductions in pregnancy-induced hypertension (OR 0.77; 95% CI 0.55, 1.07; *P *= 0.121) and risk of babies being born LGA (OR 0.86; 95% CI 0.72, 1.04; *P *= 0.117). Rates of miscarriages, preterm births, live births, or babies born small for gestational age were not impacted by weight loss.

**LIMITATIONS, REASONS FOR CAUTION:**

In our study, the intention of pregnancy was unknown. Women who intend to conceive are more likely to attempt weight loss. The inclusion of women who do not intend to conceive may therefore underestimate the true relationship between weight loss and chance of pregnancy. Also, we assessed women aged between 18 and 40 years, but due to the requirement of having two BMI records, the median age at index date across the cohort was 30 years which limits the generalizability of our findings. Furthermore, while an association between weight loss and increased chance of pregnancy was observed, we cannot imply causality as it is unknown whether higher pregnancy rates were caused by weight loss.

**WIDER IMPLICATIONS OF THE FINDINGS:**

These findings provide further evidence of the association of weight loss with reproductive outcomes in a broad population of women with overweight or obesity, including those with PCOS. Previous studies have focused mostly on outcomes in women undergoing fertility treatment. Our study was not restricted to those actively trying to conceive and, therefore, the benefit of weight loss may be even greater in women who are actively trying to become pregnant.

**STUDY FUNDING/COMPETING INTEREST(S):**

This study was funded by Novo Nordisk A/S. A.H.B. declares consultancy fees from Novo Nordisk A/S. M.L.V., V.S., E.W.L., and A.R.G. are employees of and/or hold shares in Novo Nordisk A/S. A.R.G. holds stock in Novo Nordisk. Medical writing support was provided by Carolyn Bowler, PhD, CMPP, of Apollo, OPEN Health Communications, and funded by Novo Nordisk A/S, in accordance with Good Publication Practice (GPP) guidelines (GPP 2022) (ismpp.org).

**TRIAL REGISTRATION NUMBER:**

N/A.

## Introduction

Obesity, an intricate, chronic, multifactorial disease that is a major public health challenge globally (World Health Organization, 2022), involves a complex interplay between environment/lifestyle, medical, physiological, psychosocial, and genetic factors (Gadde *et al.*, 2018; Lau and Wharton, 2020; Gjermeni *et al.*, 2021). BMI (weight in kg/height in m^2^) is the most widely used formula to measure excess weight, and people with BMI ≥25 and ≥30 kg/m^2^ are considered to have overweight and obesity, respectively (Gadde *et al.*, 2018; Lau and Wharton, 2020; Gjermeni *et al.*, 2021; World Health Organization, 2022). Excess weight is linked to multiple comorbidities such as cardiovascular disease, type 2 diabetes, respiratory conditions (e.g. asthma and obstructive sleep apnoea), impaired mobility (e.g. due to osteoarthritis), and polycystic ovary syndrome (PCOS) (Lau and Wharton, 2020; World Health Organization, 2022; Evans *et al.*, 2023).

Compared with women with BMI in the normal weight range (18.5–24.9 kg/m^2^), women with overweight or obesity face difficulties conceiving naturally (Bloom *et al.*, 2021), and are at higher risk of pregnancy complications (e.g. increased risks of miscarriage, preterm birth, gestational diabetes, or hypertensive disorders of pregnancy) and adverse childbirth-related outcomes (e.g. stillbirth, perinatal death, foetal birth defects, or being born large for gestational age [LGA]) (Guelinckx *et al.*, 2008; Torloni *et al.*, 2009; Aune *et al.*, 2014; Catalano and Shankar, 2017). Importantly, obesity negatively impacts the long-term health of not only the mother but also her offspring, with an increased risk of congenital anomalies and the possibility of metabolic disease in later life (Chandrasekaran and Neal-Perry, 2017; Kankowski *et al.*, 2022).

Fecundity refers to an individual’s ability to conceive and reproduce, while fertility is determined by the actual number of live births observed (Leridon, 2007). Lifestyle-related factors, such as an unhealthy diet and physical inactivity, may be associated with metabolic complications and can negatively affect fecundity and fertility (Leridon, 2007; Łakoma *et al.*, 2023). Weight loss has been estimated to improve conception rates among women with overweight or obesity and PCOS (Haase *et al.*, 2023), and current guidance recommends weight loss for women with overweight or obesity to improve fertility (Creanga *et al.*, 2022; NICE, 2023). However, evidence from randomized controlled trials (RCTs) and observational studies on the effect of weight loss on chance of pregnancy and pregnancy outcomes in women with overweight or obesity is conflicting (Gaskins, 2018; Hunter *et al.*, 2021; Boyle *et al.*, 2023). Previous research has been limited by small sample sizes (Gaskins, 2018; Hunter *et al.*, 2021), a focus on different questions (such as interpregnancy weight change, weight gain interval, or weight loss and weight stability in women of all weights) (Gaskins, 2018; Radin *et al.*, 2018; Lecorguillé *et al.*, 2019) or a focus on selected populations (such as those undergoing weight-loss surgery or assisted reproduction) (Patel *et al.*, 2007; Sim *et al.*, 2014a,b; Cornthwaite *et al.*, 2016; Gaskins, 2018; Hunter *et al.*, 2021).

Therefore, this cohort study used a large and unselected population from a UK real-world database to evaluate the impact of a 10–25% weight loss versus stable weight on the chance of conception, pregnancy complications, and offspring outcomes in women with BMI ≥25 kg/m^2^.

## Materials and methods

### Study design and population

This was a cohort study of patient data extracted from the UK Clinical Practice Research Datalink (CPRD) Aurum (release May 2022) database of electronic medical records (Wolf *et al.*, 2019). The Aurum pregnancy register was used to identify pregnancy outcomes, start dates, and durations. Linked Hospital Episode Statistics data (set 22 [January 2022]) were used to identify emergency caesarean sections, offspring birth weight, and the patients’ ethnicity. The socioeconomic status of the participants and the deprivation of the area where the primary-care practices are located were based on the postcode-derived 2019 English Index of Multiple Deprivation.

Women were included if they were aged 18–40 years with BMI ≥25 kg/m^2^ at index date (the earliest date after 1 January 2000, where all inclusion and exclusion criteria were fulfilled), and had ≥1 BMI record during Year 2 of the baseline period. Women were excluded if they had a hysterectomy prior to the start of follow-up, if they were pregnant during the baseline period, if there was a record of unintentional weight loss (or malignant cancer, thyroid disorder, or large limb amputation) during the baseline period or if they had an intrauterine contraceptive device (IUCD) during the baseline period.

The study consisted of a 2-year baseline period, followed by up to 3 years of follow-up (Supplementary Fig. S1). Based on their weight change during the 2-year baseline period, women were assigned to either the stable-weight (<3% weight change) or weight-loss (10–25% weight loss) cohorts. Weight change was the difference between the mean BMI during Year 2 of the baseline period and the BMI recorded at index date. During the follow-up period, differences between the weight-loss cohort and stable-weight cohorts were investigated.

This article follows the Strengthening the Reporting of Observational Studies in Epidemiology (STROBE) reporting guidelines for cohort studies (von Elm *et al.*, 2014).

### Outcomes and assessments

The primary objective was to evaluate the association between weight change and the chance of becoming pregnant (regardless of an intent to become pregnant), with the primary outcome being the first pregnancy recorded during the 3-year follow-up period, derived from the estimated pregnancy start date in the Aurum pregnancy register.

The secondary objective was to evaluate the association between weight change and risk of adverse pregnancy outcomes. Key secondary outcomes were the occurrence of miscarriage, gestational diabetes, and pregnancy-induced hypertension during pregnancy. Additional secondary outcomes were live births (i.e. fertility), preterm births (i.e. gestational age <37 weeks), emergency caesarean sections, and babies being born large or small for gestational age (LGA or SGA; LGA birth weight above the 90th percentile and SGA weight below the 10th percentile for the same gestational age, respectively).

Records were censored at the earliest of the following events: a first record of pregnancy or hysterectomy; a first prescription of contraceptives (including IUCD) or ovulation-inducing drugs; end of record (due to patient transfer or end of practice data sharing) or the follow-up period.

### Statistical analysis

Descriptive data are reported for baseline characteristics (median with quartile 1 (25%) and quartile 3 (75%)) for continuous variables and proportions [%] for categorical variables. No imputation was carried out for missing data. For smoking status, ethnicity, and socioeconomic status, an ‘unknown’ category was introduced for women where these data were not available.

#### Primary outcome

The primary outcome was analysed using a Cox proportional hazard model, with calendar time as the underlying time variable. Assumptions of proportional hazards were verified via Kaplan–Meier plots for strata of the cohort and by Schoenfeld residuals.

Weight change was represented as a categorical variable describing the two cohorts (stable weight [<3% weight change] or weight loss [10–25% weight loss]). Covariates included: BMI at index date; age at index date (20–25 [reference], 26–30, 31–35, 36–40 years); comorbidities (yes/no) including diabetes (type 1 and type 2), hypertension and PCOS; prior pregnancy record (yes/no); smoking status (non-smoker [reference], ex-smoker, current smoker, unknown smoking status); postcode-based deprivation indices for primary-care practice and patients’ residence (quintiles, with those in the top quintile representing the least deprived [reference]); ethnicity (White [reference], Black, Asian, and unknown); and frequency of general practitioner (GP) consultations during the baseline period (tertiles, with those in the first tertile having the lowest number of GP consultations [reference]).

Interaction terms between the cohort and the index-date BMI, the age category and the index-date BMI, and the age category and the covariate indicating a previous pregnancy were also included in the model for the primary outcome.

#### Exploratory analysis of the primary outcome

An exploratory analysis used the same Cox proportional hazard model as for the primary outcome to evaluate the association between the magnitude of weight loss (3–9.9%) and weight gain (3–9.9% and 10–35%) and chance of pregnancy; for this analysis, the study design was extended to include a wider range of weight loss and weight gain (Supplementary Fig. S2).

#### Secondary outcomes

For associated pregnancy complications, women with stable weight (<3% weight change) or weight loss (10–25% weight loss) who had a pregnancy during the 3-year follow-up period that lasted for at least 90 days were evaluated. Evaluations of live birth outcomes only included those who experienced pregnancy during follow-up and excluded terminations of pregnancy. This cohort was further restricted to pregnancies that ended in live birth or stillbirth for analysing the outcomes of preterm birth and emergency caesarean section. For the outcomes of LGA or SGA, the study cohort included those with birth weight available.

Logistic regression models were used to estimate the risks of outcomes. The same set of covariates as in the Cox proportional hazard model were used; only age was represented as a continuous variable. For some outcomes, quadratic terms for BMI and/or age were included when these resulted in a significant improvement of the model. No interaction terms between any covariates were used in the logistic regression models, and the quality of the logistic regression models was assessed using area under the receiver operating characteristic curve.

All statistical analyses were carried out using the R environment for statistical computing and visualization (version 4.2.2).

### Role of the funding source

Novo Nordisk A/S is the study sponsor, and was responsible for the study design, preparing the study protocol and statistical analysis plan, and performing the statistical analyses. This article was drafted under the guidance of the authors, with medical writing and editorial support paid for by the funder.

## Results

### Baseline characteristics

#### Primary analysis cohort

The final cohort of 246 670 women comprised 195 666 who kept stable weight and 51 004 who had lost weight during the baseline period; median follow-up was ∼1 month longer in women with stable weight versus weight loss (191 [quartiles 1, 3; 68, 943] versus 166 [quartiles 1, 3; 63, 794] days) (Table 1 and Supplementary Fig. S3).

**Table 1.** Demographics and baseline characteristics in the primary analysis cohort.

	Total	**Stable weight** ^a^	**Weight loss** ^b^
	(N = 246 670)	(N = 195 666)	(N = 51 004)
***Variable, median (IQR)***			
**Age at index date, years**	30 (24, 35)	30 (24, 36)	28 (23, 34)
**BMI at index date, kg/m^2^**	28.6 (26.2, 33.1)	28.4 (26.1, 32.8)	29.4 (26.7, 34.0)
**BMI at Year 2, kg/m^2^**	27.8 (25.7, 32.2)	28.4 (26.2, 32.9)	25.1 (23.0, 29.0)
**Weight change, %**	−0.4 (−2.6, 1.1)	0.0 (−1.3, 1.4)	−13.6 (−16.8, −11.5)
**Follow-up, days**	185 (67, 912)	191 (68, 943)	166 (63, 794)
***Weight category, n (%)***			
**Overweight (BMI 25–29.9 kg/m^2^)**	146 899 (59.6)	119 357 (61.0)	27 542 (54.0)
**Obesity I (BMI 30–34.9 kg/m^2^)**	54 326 (22.0)	41 742 (21.3)	12 584 (24.7)
**Obesity II (BMI 35–39.9 kg/m^2^)**	26 248 (10.6)	20 190 (10.3)	6058 (11.9)
**Obesity III (BMI ≥40 kg/m^2^)**	19 197 (7.8)	14 377 (7.3)	4820 (9.5)
***Smoking status, n (%)***			
**Current**	53 238 (21.6)	40 001 (20.4)	13 237 (26.0)
**Ex**	42 920 (17.4)	33 610 (17.2)	9310 (18.3)
**Never**	125 861 (51.0)	102 460 (52.4)	23 401 (45.9)
**Unknown**	24 651 (10.0)	19 595 (10.0)	5056 (9.9)
***Comorbidities at start of follow-up, n (%)***			
**Diabetes (overall)**	10 002 (4.1)	8162 (4.2)	1840 (3.6)
**T1D**	2580 (1.1)	2069 (1.1)	511 (1.0)
**T2D**	7422 (3.0)	6093 (3.1)	1329 (2.6)
**Hypertension**	13 885 (5.6)	11 511 (5.9)	2374 (4.7)
**PCOS**	16 100 (6.5)	12 811 (6.5)	3289 (6.4)
**Pregnancy before index date**	118 700 (48.1)	92 628 (47.3)	26 072 (51.1)
***Outcome/reason for censoring, n (%)***			
**Pregnancy during follow-up**	22 756 (9.2)	17 650 (9.0)	5106 (10.0)
**Censored due to end of record**	36 387 (14.8)	29 086 (14.9)	7301 (14.3)
**Censored due to contraceptives/IUCD**	132 765 (53.8)	104 426 (53.4)	28 339 (55.6)
**Censored due to prescription of ovulation-inducing drugs**	433 (0.2)	360 (0.2)	73 (0.1)

a <3% weight change.

b 10–25% weight loss.

BMI, body mass index; IQR, interquartile range; PCOS, polycystic ovary syndrome; T1D, type 1 diabetes; T2D, type 2 diabetes; IUCD, intrauterine contraceptive device.

Aside from age, starting BMI, hypertension, smoking status, and parity, baseline characteristics were generally balanced across the two cohorts, including the prevalence of PCOS and diabetes (Table 1 and Supplementary Table S1). Women in the weight-loss cohort were younger than those with stable weight (median age: 28 years versus 30 years, respectively; *P *< 0.001). Notably, median BMI in Year 1 was higher in women with weight loss versus stable weight (29.4 kg/m^2^ versus 28.4 kg/m^2^; *P *< 0.001). For women with stable weight versus weight loss, slightly more had hypertension (5.9% versus 4.7%; *P *< 0.001), more were never smokers (52.4% versus 45.9%; *P *< 0.001), and fewer had a prior pregnancy (47.3% versus 51.1%; *P *< 0.001) or were current smokers (20.4% versus 26.0%; *P *< 0.001). Pregnancy during follow-up occurred in 9.2% versus 10.3% of women with stable weight versus weight loss (*P *= 0.08). Most (77.8%) women were not followed for the full 3 years, with the primary reason being prescription of contraceptives (53.8% of women).

#### Secondary analysis cohort

Characteristics of women who had a pregnancy during the 3-year follow-up period that lasted for at least 90 days and were evaluated for associated pregnancy complications are shown in Table 2 and Supplementary Table S2. Median follow-up was similar between women with stable weight versus weight loss (249 [quartiles 1, 3; 109, 495] versus 232 [quartiles 1, 3; 100, 474] days, respectively), and aside from weight change and BMI, baseline characteristics were generally balanced across the cohorts, with similar patterns to the primary analysis cohort (Table 2 and Supplementary Table S2). Median BMI in Year 1 was higher in women with weight loss versus stable weight (29.7 kg/m^2^ versus 28.5 kg/m^2^), with a higher proportion in each obesity weight category versus stable weight (e.g. 10.3% versus 6.9% for obesity III).

**Table 2.** Characteristics of women evaluated for associations with pregnancy complications (those who had a pregnancy during the 3-year follow-up period that lasted for at least 90 days).

	Total	**Stable weight** ^a^	**Weight loss** ^b^
	(N = 12 224)	(N = 9452)	(N = 2772)
***Variable, median (IQR)***			
**Age at index date, years**	28 (24, 32)	28 (25, 32)	28 (24, 31)
**BMI at index date, kg/m^2^**	28.8 (26.3, 33.2)	28.5 (26.2, 32.9)	29.7 (26.9, 34.1)
**BMI at Year 2, kg/m^2^**	28.0 (25.8, 32.2)	28.6 (26.3, 32.9)	25.4 (23.1, 29.2)
**Median weight change, %**	−0.6 (−2.8, 1.1)	0.0 (−1.3, 1.5)	−13.8 (−16.7, −11.6)
**Median follow-up, days**	245 (106, 489)	249 (109, 495)	232 (100, 474)
***Weight category, n (%)***			
**Overweight (BMI 25–29.9 kg/m^2^)**	7108 (58.1)	5674 (60.0)	1434 (51.7)
**Obesity I (BMI 30–34.9 kg/m^2^)**	2826 (23.1)	2110 (22.3)	716 (25.8)
**Obesity II (BMI 35–39.9 kg/m^2^)**	1351 (11.1)	1014 (10.7)	337 (12.2)
**Obesity III (BMI ≥40 kg/m^2^)**	939 (7.7)	654 (6.9)	285 (10.3)
***Smoking status, n (%)***			
**Current**	2709 (22.2)	1963 (20.8)	746 (26.9)
**Ex**	2235 (18.3)	1670 (17.7)	565 (20.4)
**Never**	5988 (49.0)	4802 (50.8)	1186 (42.8)
**Unknown**	1292 (10.6)	1017 (10.8)	275 (9.9)
***Comorbidities at start of follow-up, n (%)***			
**Diabetes (overall)**	399 (3.3)	311 (3.3)	88 (3.2)
**T1D**	141 (1.2)	110 (1.2)	31 (1.1)
**T2D**	258 (2.1)	201 (2.1)	57 (2.1)
**Hypertension**	484 (4.0)	383 (4.1)	101 (3.6)
**PCOS**	1095 (9.0)	850 (9.0)	245 (8.8)
**Pregnancy before index date**	6963 (57.0)	5176 (54.8)	1787 (64.5)

a <3% weight change.

b 10–25% weight loss.

BMI, body mass index; IQR, interquartile range; PCOS, polycystic ovary syndrome; T1D, type 1 diabetes; T2D, type 2 diabetes.

### Outcomes

Table 3 provides an overview of the events for the primary and secondary analyses.

**Table 3.** The size of the study cohorts and the number of events for the primary and secondary objectives.

	Event	Number at risk (complete cases)	Number of events	Ratio
**Primary outcome**	Pregnancy	246 670	22 756	9.2%
**Pregnancy complications**	Miscarriage	22 756	2463	10.8%
	Gestational diabetes	11 825	950	8.0%
	Pregnancy-induced hypertension	11 740	244	2.1%
**Pregnancy outcomes**	Live birth	14 739	11 515	78.1%
	Emergency caesarean section	11 558	1453	12.6%
	Preterm birth	11 558	561	4.9%
	Small for gestational age	7988	1432	17.9%
	Large for gestational age	7988	791	9.9%

#### Weight change and the chance of pregnancy

**Figure 1.**
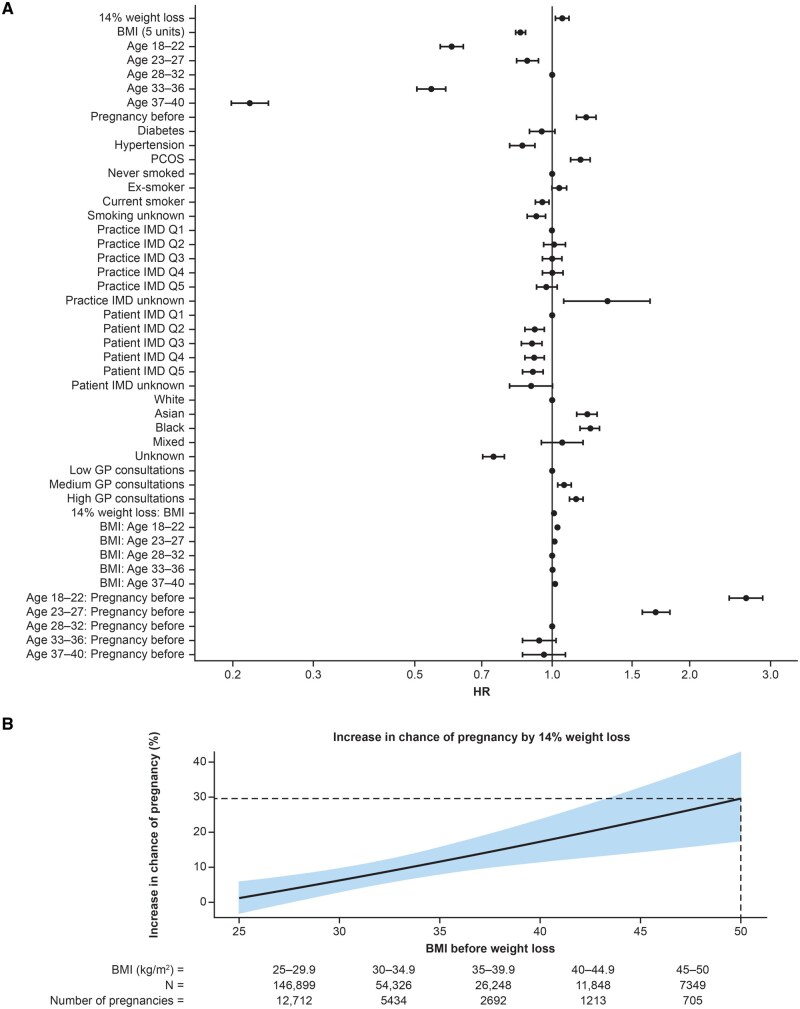
**Primary outcome of a first pregnancy recorded during the 3-year follow-up period**: (**A**) Forest plot describing the contributions of the individual covariates for the primary outcome; (**B**) estimated increase in the chance of pregnancy in the weight-loss^a^ cohort in comparison to the stable-weight^b^ cohort across the BMI range. ^a^10–25% weight loss. ^b^<3% weight change. GP, general ractitioner; HR, hazard ratio; IMD, Index of Multiple Deprivation; PCOS, polycystic ovary syndrome; Q, quintile.

**Figure 2.**
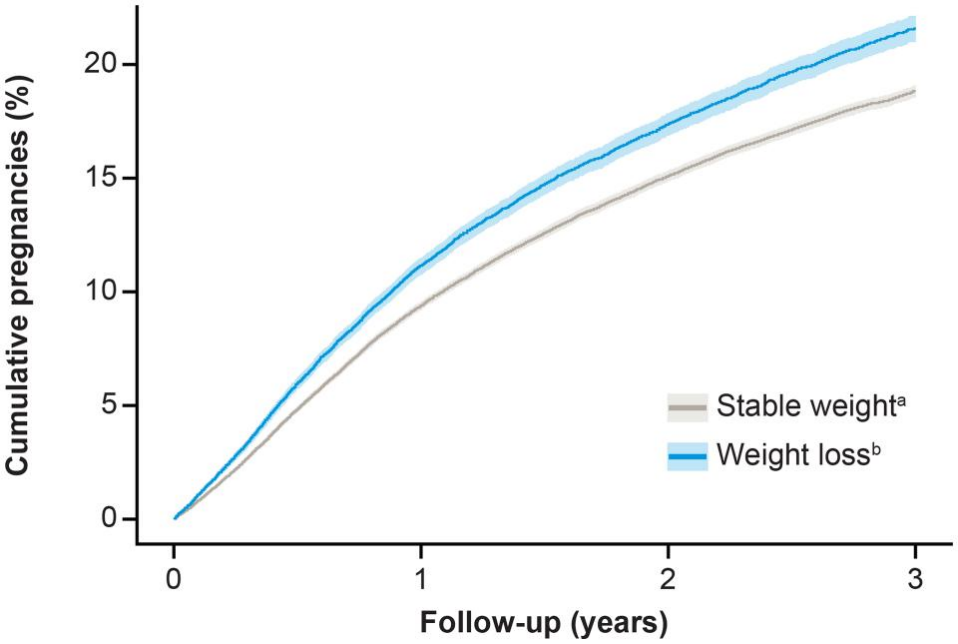
**Predicted cumulative single pregnancies by age group if all participants were followed for the full 3 years.** Data shown are for single pregnancies only. Women could not contribute data from a second pregnancy unless they switched practice and received a second patient ID, and were then included in the study under that second ID, which was considered unlikely. ^a^<3% weight change. ^b^10–25% weight loss.

Overall, the median weight loss in the 10–25% weight loss cohort was 14%. The average woman in the weight-loss cohort (median BMI 29.0 kg/m^2^) had a 5.2% increase in the chance of pregnancy compared to the average woman in the stable-weight cohort (hazard ratio [HR] 1.05; 95% CI 1.02, 1.09; *P *= 0.003) (Fig. 1A and Supplementary Table S3). Furthermore, this increase was greatest among women with higher baseline BMI; for example, a 14% median weight loss in a woman with BMI of 45 kg/m^2^ before weight loss increased the chance of pregnancy by 23% (HR 1.23; 95% CI 1.14, 1.33; *P *< 0.001) (Fig. 1B). Conversely, a 5-unit-higher BMI at index date was associated with a 15% lower chance of pregnancy (HR 0.85; 95% CI 0.83, 0.87; *P *< 0.001) (Supplementary Table S3). Pre-existing baseline hypertension and PCOS were associated with a lower (HR 0.86; 95% CI 0.81, 0.92; *P *< 0.001) and a higher (HR 1.16; 95% CI 1.10, 1.21; *P *< 0.001) chance of pregnancy, respectively, while the presence of diabetes (including both type 1 and type 2) had no impact (HR 0.95; 95% CI 0.89, 1.01) (Supplementary Table S3). Significant interactions were observed for 14% weight loss and BMI at index-date, age category (18–22, 23–27, and 37–40) and BMI at index date, and age category (18–22, and 23–27) and previous pregnancy (all *P *< 0.001) (Supplementary Table S3). The overlap between the proportion of women with diabetes, hypertension, and PCOS at baseline is shown in Supplementary Fig. S4. The predicted cumulative frequency of single pregnancies in women with stable weight and weight loss, if all participants were followed for the full 3 years, is shown in Fig. 2.

Supplementary Figure S5 shows outcomes from the exploratory analysis of the association between the magnitude of weight change and the chance of pregnancy. The chance of pregnancy increased in women with 10–25% weight loss (HR 1.05; 95% CI 1.02, 1.09; *P *= 0.002) and 3–9.9% weight loss (HR 1.03; 95% CI 1.01, 1.06; *P *= 0.01) (Supplementary Fig. S5A). However, the chance of pregnancy decreased in women with 3–9.9% weight gain (HR 0.97; 95% CI 0.95, 0.99; *P *= 0.02) and 10–25% weight gain (HR 0.92; 95% CI 0.89, 0.95; *P *< 0.001). Correspondingly, an average 5-unit increase in starting BMI was associated with a 3% reduction in the rate of pregnancy (HR 0.97; 95% CI 0.95, 0.99; *P = *0.01) (Supplementary Fig. S5A). While baseline BMI did not impact the chance of pregnancy in women with lower weight loss (3–9.9%), higher baseline BMI was associated with a decrease in the chance of pregnancy in women with weight gain (3–9.9% and 10–25%) (Supplementary Fig. S5B).

#### Weight loss and the risk of pregnancy complications

**Figure 3.**
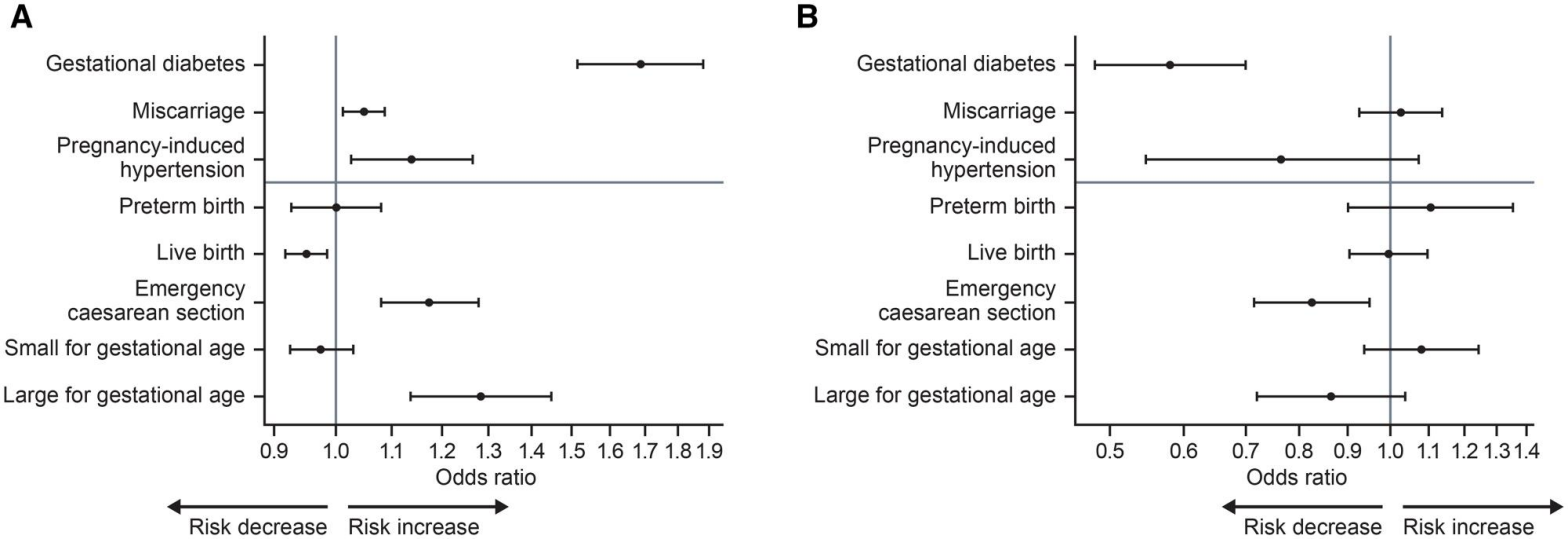
**Association between covariates and secondary outcomes.** (**A**) 5-unit-higher baseline BMI. (**B**) 14% weight loss.

Associations between weight loss and the risk of pregnancy complications during the 3-year follow-up are shown in Fig. 3, Supplementary Fig. S6, and Supplementary Table S4. For every 5-unit increase in baseline BMI, the risk of gestational diabetes increased by ∼69% (odds ratio [OR] 1.69; 95% CI 1.52, 1.88; *P *< 0.001), pregnancy-induced hypertension increased by ∼14% (OR 1.14; 95% CI 1.03, 1.26; *P *= 0.01), and miscarriage increased by ∼5% (OR 1.05; 95% CI 1.01, 1.09; *P *= 0.008). A 14% weight loss reduced the risk of gestational diabetes by 42% (OR 0.58; 95% CI 0.48, 0.70; *P *< 0.001), and while there was a reduction in pregnancy-induced hypertension, this was not statistically significant (OR 0.77; 95% CI 0.55, 1.07) and there was no effect on the risk of miscarriage (OR 1.03; 95% CI 0.93, 1.14). A previous diagnosis of PCOS was associated with a significantly higher risk of gestational diabetes (OR 1.50; 95% CI 1.23, 1.83; *P *< 0.001), miscarriage (OR 1.20; 95% CI 1.05, 1.38; *P *= 0.009), and pregnancy-induced hypertension (OR 1.56; 95% CI 1.05, 2.29; *P *= 0.02).

#### Weight loss and adverse pregnancy outcomes

Associations between weight loss and the risk of adverse pregnancy outcomes during the 3-year follow-up are shown in Fig. 3, Supplementary Fig. S7, and Supplementary Table S5. Women with a 14% weight loss had a significant risk reduction in emergency caesarean section (OR 0.82; 95% CI 0.71, 0.95; *P *= 0.008). For every 5-unit increase in baseline BMI, the risk of emergency caesarean section increased by ∼17% (OR 1.17; 95% CI 1.08, 1.28; *P *< 0.001), delivering an LGA baby increased by ∼28% (OR 1.28; 95% CI 1.14, 1.45; *P *< 0.001) and the chance of a live birth decreased by ∼5% (OR 0.95; 95% CI 0.92, 0.99; *P *= 0.005).

## Discussion

In this cohort of women with overweight or obesity for whom pregnancy intention was unknown, we observed that women had an improved chance of pregnancy in the 3 years following weight loss, compared with those who maintained their weight. A median 14% weight loss was associated with a small (5.2%) but statistically significant increase in the chance of pregnancy. However, the chance of pregnancy was 17% and 23% higher in women with a baseline BMI of 40 and 45 kg/m^2^ who had a 14% weight loss during the 2-year baseline period compared with those who maintained their body weight.

These data are an important addition to the literature on the topic of weight loss and reproductive outcomes. The majority of published data focuses upon the benefits of weight loss in women undergoing fertility treatment, rather than in general populations where there would be women in a mixture of circumstances (i.e. natural conceptions and those actively trying to conceive) (Gaskins, 2018; Hunter *et al.*, 2021). As far as we are aware, there are no other large-scale observational studies that have evaluated the effect of weight loss on the chance of pregnancy and pregnancy outcomes for women with obesity in general populations. Few RCTs have reported the effects of weight loss on pregnancy directly; instead, drop-outs due to pregnancy have been used to evaluate the potential association of weight loss with pregnancy (Boyle *et al.*, 2023). Moreover, RCTs have included women with obesity and known infertility, or conditions known to affect fertility (e.g. PCOS) (Sim *et al.*, 2014a,b; Gaskins, 2018; Hunter *et al.*, 2021). Our results complement those reported from a cohort of women recently diagnosed with PCOS and a baseline BMI of 40 kg/m^2^, where a 10% weight loss was associated with an increase in the chance of pregnancy of 68% (Haase *et al.*, 2023). There are several reasons why the chance of pregnancy differed between our study and the PCOS study. While PCOS affects <20% of women, it accounts for around 80% of anovulatory infertility (Balen *et al.*, 2016). Thus, the 68% increase reported by Haase *et al.* is from a PCOS population that has a lower baseline pregnancy rate. Despite our population including women with or without PCOS, who may or may not have been actively trying to become pregnant, weight loss increased the chance of pregnancy by 5%. In addition, the index dates differed between studies. The index date in the PCOS study was the date of PCOS diagnosis (Haase *et al.*, 2023), while in our study, a 2-year baseline period marked the index date (the earliest BMI record at adult age where all eligibility criteria were fulfilled). Importantly, in the PCOS study, women might have received a PCOS diagnosis while actively trying to become pregnant (Haase *et al.*, 2023).

It is unknown if women in our study were actively trying to become pregnant. However, we did observe that in our general population, women with PCOS had a 15% higher chance of pregnancy than those without PCOS (for the reasons discussed above), although there was also a tendency for women with PCOS to have more miscarriages, preterm births, and emergency caesarean sections, and fewer live births. Recent International PCOS Network guidance states that healthcare professionals should be aware that women with PCOS are at a higher risk of miscarriage than those without PCOS (Teede *et al.*, 2023). Indeed, the International PCOS Network recommends that women with PCOS receiving fertility treatment should be counselled on the adverse impact of excess weight on clinical pregnancy, miscarriage, and live birth rates (Teede *et al.*, 2023).

We observed a large and significant interaction between young maternal age and previous pregnancy. Compared with the refence group (28–32 years), women aged 18–22 years had a 40% lower chance of pregnancy unless they have had a previous pregnancy, in which case the chance of pregnancy more than doubled.

While we did not observe a significant benefit of weight loss for most of our secondary outcomes, we did observe that weight loss significantly reduced the risk of gestational diabetes (42% risk reduction) and emergency caesarean section (18% risk reduction). A meta-analysis of observational studies has reported significant associations between interpregnancy weight loss and gestational diabetes (25% risk reduction) and emergency caesarean section (12% risk reduction for BMI ≥25 kg/m^2^) (Nagpal *et al.*, 2022). In our study, it appears that in women with weight loss, the significant reduction in emergency caesarean sections may be driven mainly by fewer LGA babies being born. Conversely, women with a higher baseline BMI had an increased risk of emergency caesarean sections and were more likely to give birth to LGA babies.

We did not observe any benefit of weight loss upon the rate of miscarriage in our general population. Similarly, a US prospective cohort study that looked at the risk of pregnancy loss in women with weight change (half were with overweight or obesity at baseline) during the 12–18 months before natural conception found that attempted or achieved weight loss had no impact upon the miscarriage rate (Radin *et al.*, 2018). In contrast, Clark *et al.* (1998) reported that weight loss decreased the miscarriage rate in the same group of women with obesity undergoing fertility treatment (18% [10/55 pregnancies] after weight loss versus 75% [6/8 pregnancies] before weight loss), although the pregnancy numbers before weight loss were small.

We also did not detect any benefit of weight loss upon the rates of preterm or live births. A meta-analysis of prospective and retrospective observational studies reported that interpregnancy weight loss in women with overweight increased the risk of preterm birth by 6% (Nagpal *et al.*, 2022). A systematic review reported an increase in live births in 7/11 studies evaluating weight loss in women with overweight or obesity undergoing fertility treatment, although the quality of the studies was weak (Sim *et al.*, 2014b). In addition, the number of live births has been reported to increase in women with obesity undergoing fertility treatment who did versus did not lose weight (by 44% with −6.6 kg versus 14% with −1.6 kg weight loss (Sim *et al.*, 2014a); 71% versus 37% in those with versus those without ≥10% weight loss (Kort *et al.*, 2014)). However, a recent retrospective review and meta-analysis of women with overweight or obesity found that significant weight loss before initiating fertility treatment did not improve the live birth rate versus those without weight loss (mean difference: −4.62 kg; 95% CI −8.11, −1.14; OR 1.38; 95% CI 0.88, 2.10) (Jeong *et al.*, 2024). In contrast, weight loss before pregnancy in women with overweight reduced the risk of neonatal mortality (Cnattingius and Villamor, 2016).

Finally, we did not observe any benefit of weight loss upon the rates of SGA and LGA babies. In an analysis of data from the Étude Longitudinale Française depuis l’Enfance (ELFE) French national birth cohort, weight loss of >5 kg in women with overweight or obesity the year before pregnancy did not affect birth weight (Lecorguillé *et al.*, 2019). However, in these women, mediation analyses found that gestational weight gain cancelled out the effect of weight loss before pregnancy on reduced infant birth weight (Lecorguillé *et al.*, 2019). Furthermore, a systematic review reported that interpregnancy weight loss increased the risk of SGA babies by 29% (Nagpal *et al.*, 2022). Interestingly, while the odds of giving birth to SGA babies increased significantly in women with BMI <25 kg/m^2^ by 49%, this was not seen in women with BMI ≥25 kg/m^2^ (Nagpal *et al.*, 2022). Taken together, our findings support the known associations of increased body weight with many of these outcomes, even when there was no benefit of weight loss.

### Strengths and limitations

A strength of our study is that we observed an association between weight loss and higher chance of conception and reduced risk of gestational diabetes and emergency caesarean section in a large, nationally representative real-world population, although we cannot definitively state a causal relationship. One limitation from a modelling perspective is that our study was not restricted to women who were actively trying to become pregnant during follow-up. This could lead to potential confounding—for instance, weight loss may be an indication of an intent to become pregnant, which could mean that there were more women with the intention to become pregnant in the weight-loss group than in the stable-weight group. It is also possible that, because of the potential use of contraceptives, the benefit of weight loss in a population actively trying to conceive might be even greater. Other limitations include that records were censored only when contraceptives were prescribed during follow-up; however, women may have used non-prescription-based contraceptives, the use of which could have differed between weight-change groups. Although we used the earliest BMI record as the index date for adult women, the median age at index date across the cohort was 30 years. This was likely due to the inclusion criterion of two BMI records during the baseline period, which was only available for ∼20% of the women who had a BMI measurement between 18 and 40 years of age, limiting the generalizability of our findings to this group. Lastly, our sample size was too small to evaluate the occurrence of congenital defects as an outcome.

## Conclusions

Although there is extensive evidence on the impact of weight loss focused on women undergoing fertility treatment and its outcomes, this is the first study to report the effect of weight loss on the chance of pregnancy and pregnancy outcomes in a general population of women with overweight or obesity, who may or may not have been trying to get pregnant. In this real-world population of women with overweight or obesity, weight loss was associated with a small increase in the chance of pregnancy over a 3-year period. Of the women who became pregnant, those who lost weight had a statistically significant reduction in the risk of gestational diabetes and emergency caesarean section, and a non-statistically significant reduction in pregnancy-induced hypertension, with fewer LGA babies being born compared with women of stable weight. However, there was no association between weight loss and preterm birth, live birth, miscarriage, or SGA babies. These findings provide further evidence of the benefits of weight loss on chance of pregnancy and pregnancy-related complications among women with overweight or obesity. In the future, retrospective study designs may result in larger cohorts, and higher power to detect any relevant associations between pre-pregnancy body weight, or body-weight changes, and pregnancy complications.

## Supplementary Material

deaf122_Supplementary_Figure_S1

deaf122_Supplementary_Figure_S2

deaf122_Supplementary_Figure_S3

deaf122_Supplementary_Figure_S4

deaf122_Supplementary_Figure_S5

deaf122_Supplementary_Figure_S6

deaf122_Supplementary_Figure_S7

deaf122_Supplementary_Table_S1

deaf122_Supplementary_Table_S2

deaf122_Supplementary_Table_S3

deaf122_Supplementary_Table_S4

deaf122_Supplementary_Table_S5

## Data Availability

This study is based on data from the CPRD obtained under licence from the UK Medicines and Healthcare products Regulatory Agency. The data are provided by patients and collected by the UK National Health Service as part of their care and support. The interpretation and conclusions contained in this study are those of the authors alone. Electronic health records are classified as ‘sensitive data’ by the UK Data Protection Act; therefore, information governance restrictions prevent data sharing via public deposition. Information about access to CPRD data is available here: https://www.cprd.com/research-applications.

## References

[deaf122-B1] Aune D , Saugstad OD, Henriksen T, Tonstad S. Maternal body mass index and the risk of fetal death, stillbirth, and infant death: a systematic review and meta-analysis. JAMA 2014;311:1536–1546. 24737366 10.1001/jama.2014.2269

[deaf122-B2] Balen AH , Morley LC, Misso M, Franks S, Legro RS, Wijeyaratne CN, Stener-Victorin E, Fauser BC, Norman RJ, Teede H. The management of anovulatory infertility in women with polycystic ovary syndrome: an analysis of the evidence to support the development of global WHO guidance. Hum Reprod Update 2016;22:687–708. 27511809 10.1093/humupd/dmw025

[deaf122-B3] Bloom MS , Perkins NJ, Sjaarda LA, Mumford SL, Ye A, Kim K, Kuhr DL, Nobles CJ, Connell MT, Schisterman EF. Adiposity is associated with anovulation independent of serum free testosterone: a prospective cohort study. Paediatr Perinat Epidemiol 2021;35:174–183. 33107110 10.1111/ppe.12726 PMC7878298

[deaf122-B4] Boyle BR , Ablett AD, Ochi C, Hudson J, Watson L, Rauh D, Avenell A. The effect of weight loss interventions for obesity on fertility and pregnancy outcomes: a systematic review and meta-analysis. Int J Gynaecol Obstet 2023;161:335–342. 36440496 10.1002/ijgo.14597

[deaf122-B5] Catalano PM , Shankar K. Obesity and pregnancy: mechanisms of short term and long term adverse consequences for mother and child. BMJ 2017;356:j1. 28179267 10.1136/bmj.j1 PMC6888512

[deaf122-B6] Chandrasekaran S , Neal-Perry G. Long-term consequences of obesity on female fertility and the health of the offspring. Curr Opin Obstet Gynecol 2017;29:180–187. 28448277 10.1097/GCO.0000000000000364 PMC5983896

[deaf122-B7] Clark AM , Thornley B, Tomlinson L, Galletley C, Norman RJ. Weight loss in obese infertile women results in improvement in reproductive outcome for all forms of fertility treatment. Hum Reprod 1998;13:1502–1505. 9688382 10.1093/humrep/13.6.1502

[deaf122-B8] Cnattingius S , Villamor E. Weight change between successive pregnancies and risks of stillbirth and infant mortality: a nationwide cohort study. Lancet 2016;387:558–565. 26651225 10.1016/S0140-6736(15)00990-3

[deaf122-B9] Cornthwaite K , Jefferys A, Lenguerrand E, Haase A, Lynch M, Johnson A, Draycott T, Siassakos D. Pregnancy after weight loss surgery: a commentary. BJOG 2016;123:165–170. 26841364 10.1111/1471-0528.13791

[deaf122-B10] Creanga AA , Catalano PM, Bateman BT. Obesity in pregnancy. N Engl J Med 2022;387:248–259. 35857661 10.1056/NEJMra1801040

[deaf122-B11] Evans M , de Courcy J, de Laguiche E, Faurby M, Haase CL, Matthiessen KS, Moore A, Pearson-Stuttard J. Obesity-related complications, healthcare resource use and weight loss strategies in six European countries: the RESOURCE survey. Int J Obes (Lond) 2023;47:750–757. 37258646 10.1038/s41366-023-01325-1 PMC10359184

[deaf122-B13] Gadde KM , Martin CK, Berthoud H-R, Heymsfield SB. Obesity: pathophysiology and management. J Am Coll Cardiol 2018;71:69–84. 29301630 10.1016/j.jacc.2017.11.011 PMC7958889

[deaf122-B14] Gaskins AJ. Recent advances in understanding the relationship between long- and short-term weight change and fertility. F1000Res 2018;7:F1000 Faculty Rev-1702. 10.12688/f1000research.15278.1 PMC6206616 30416711

[deaf122-B15] Gjermeni E , Kirstein AS, Kolbig F, Kirchhof M, Bundalian L, Katzmann JL, Laufs U, Blüher M, Garten A, Le Duc D. Obesity—an update on the basic pathophysiology and review of recent therapeutic advances. Biomolecules 2021;11:1426. 34680059 10.3390/biom11101426 PMC8533625

[deaf122-B16] Guelinckx I , Devlieger R, Beckers K, Vansant G. Maternal obesity: pregnancy complications, gestational weight gain and nutrition. Obes Rev 2008;9:140–150. 18221480 10.1111/j.1467-789X.2007.00464.x

[deaf122-B17] Haase CL , Varbo A, Laursen PN, Schnecke V, Balen AH. Association between body mass index, weight loss and the chance of pregnancy in women with polycystic ovary syndrome and overweight or obesity: a retrospective cohort study in the UK. Hum Reprod 2023;38:471–481. 36637246 10.1093/humrep/deac267 PMC9977115

[deaf122-B18] Hunter E , Avenell A, Maheshwari A, Stadler G, Best D. The effectiveness of weight-loss lifestyle interventions for improving fertility in women and men with overweight or obesity and infertility: a systematic review update of evidence from randomized controlled trials. Obes Rev 2021;22:e13325. 34390109 10.1111/obr.13325

[deaf122-B19] Jeong HG , Cho S, Ryu KJ, Kim T, Park H. Effect of weight loss before in vitro fertilization in women with obesity or overweight and infertility: a systematic review and meta-analysis. Sci Rep 2024;14:6153. 38486057 10.1038/s41598-024-56818-4 PMC10940611

[deaf122-B20] Kankowski L , Ardissino M, McCracken C, Lewandowski AJ, Leeson P, Neubauer S, Harvey NC, Petersen SE, Raisi-Estabragh Z. The impact of maternal obesity on offspring cardiovascular health: a systematic literature review. Front Endocrinol (Lausanne) 2022;13:868441. 35669689 10.3389/fendo.2022.868441 PMC9164814

[deaf122-B21] Kort JD , Winget C, Kim SH, Lathi RB. A retrospective cohort study to evaluate the impact of meaningful weight loss on fertility outcomes in an overweight population with infertility. Fertil Steril 2014;101:1400–1403. 24581574 10.1016/j.fertnstert.2014.01.036

[deaf122-B22] Łakoma K , Kukharuk O, Śliż D. The influence of metabolic factors and diet on fertility. Nutrients 2023;15:1180. 36904180 10.3390/nu15051180 PMC10005661

[deaf122-B23] Lau DCW , Wharton S. *Canadian Adult Obesity Clinical Practice Guidelines: the science of obesity.* Ottawa, Ontario, Canada: Canadian Medical Association, 2020. https://obesitycanada.ca/guidelines/science

[deaf122-B24] Lecorguillé M , Jacota M, de Lauzon-Guillain B, Forhan A, Cheminat M, Charles MA, Heude B. An association between maternal weight change in the year before pregnancy and infant birth weight: ELFE, a French national birth cohort study. PLoS Med 2019;16:e1002871. 31430274 10.1371/journal.pmed.1002871 PMC6701747

[deaf122-B25] Leridon H. Studies of fertility and fecundity: comparative approaches from demography and epidemiology. C R Biol 2007;330:339–346. 17502290 10.1016/j.crvi.2007.02.013

[deaf122-B26] Nagpal TS , Souza SCS, Moffat M, Hayes L, Nuyts T, Liu RH, Bogaerts A, Dervis S, Piccinini-Vallis H, Adamo KB et al Does prepregnancy weight change have an effect on subsequent pregnancy health outcomes? A systematic review and meta-analysis. Obes Rev 2022;23:e13324. 34694053 10.1111/obr.13324

[deaf122-B27] NICE. *Weight management before, during and after pregnancy*. London, UK: National Institute for Health and Care Excellence (NICE), 2023. https://www.nice.org.uk/guidance/ph27/resources/weight-management-before-during-and-after-pregnancy-pdf-1996242046405

[deaf122-B29] Patel JA , Colella JJ, Esaka E, Patel NA, Thomas RL. Improvement in infertility and pregnancy outcomes after weight loss surgery. Med Clin North Am 2007;91:515–528, xiii. 17509393 10.1016/j.mcna.2007.01.002

[deaf122-B30] Radin RG , Mumford SL, Sjaarda LA, Silver RM, Wactawski-Wende J, Lynch AM, Perkins NJ, Lesher LL, Wilcox BD, Hinkle SN et al Recent attempted and actual weight change in relation to pregnancy loss: a prospective cohort study. BJOG 2018;125:676–684. 29067752 10.1111/1471-0528.14859 PMC5918461

[deaf122-B31] Sim KA , Dezarnaulds GM, Denyer GS, Skilton MR, Caterson ID. Weight loss improves reproductive outcomes in obese women undergoing fertility treatment: a randomized controlled trial. Clin Obes 2014a;4:61–68. 25826729 10.1111/cob.12048

[deaf122-B32] Sim KA , Partridge SR, Sainsbury A. Does weight loss in overweight or obese women improve fertility treatment outcomes? A systematic review. Obes Rev 2014b;15:839–850. 25132280 10.1111/obr.12217

[deaf122-B33] Teede HJ , Tay CT, Laven J, Dokras A, Moran LJ, Piltonen TT, Costello MF, Boivin J, Redman LM, Boyle JA et al; International PCOS Network. Recommendations from the 2023 International Evidence-based Guideline for the Assessment and Management of Polycystic Ovary Syndrome^†^. Hum Reprod 2023;38:1655–1679. 37580037 10.1093/humrep/dead156 PMC10477934

[deaf122-B34] Torloni MR , Betrán AP, Daher S, Widmer M, Dolan SM, Menon R, Bergel E, Allen T, Merialdi M. Maternal BMI and preterm birth: a systematic review of the literature with meta-analysis. J Matern Fetal Neonatal Med 2009;22:957–970. 19900068 10.3109/14767050903042561

[deaf122-B35] von Elm E , Altman DG, Egger M, Pocock SJ, Gøtzsche PC, Vandenbroucke JP; STROBE Initiative. The Strengthening the Reporting of Observational Studies in Epidemiology (STROBE) Statement: guidelines for reporting observational studies. Int J Surg 2014;12:1495–1499.25046131

[deaf122-B36] Wolf A , Dedman D, Campbell J, Booth H, Lunn D, Chapman J, Myles P. Data resource profile: Clinical Practice Research Datalink (CPRD) Aurum. Int J Epidemiol 2019;48:1740–1740g. 30859197 10.1093/ije/dyz034 PMC6929522

[deaf122-B37] World Health Organization. *WHO European Regional Obesity Report 2022*. Copenhagen, Denmark: World Health Organization, 2022. https://iris.who.int/bitstream/handle/10665/353747/9789289057738-eng.pdf

