# Supplementary material for: Association between weight loss and reproductive outcomes among women with overweight or obesity: a cohort study using UK real-world data
Source: Hum Reprod. 2025 Jul 6;40(9):1753–61. doi: 10.1093/humrep/deaf122 (PMC12408893; doi:10.1093/humrep/deaf122)
Supplement: deaf122_Supplementary_Figure_S6 [file deaf122_supplementary_figure_s6.pdf]

Human Reproduction, 2025, 1–1
https://doi.org/10.1093/humrep/deaf122
Supplementary Data

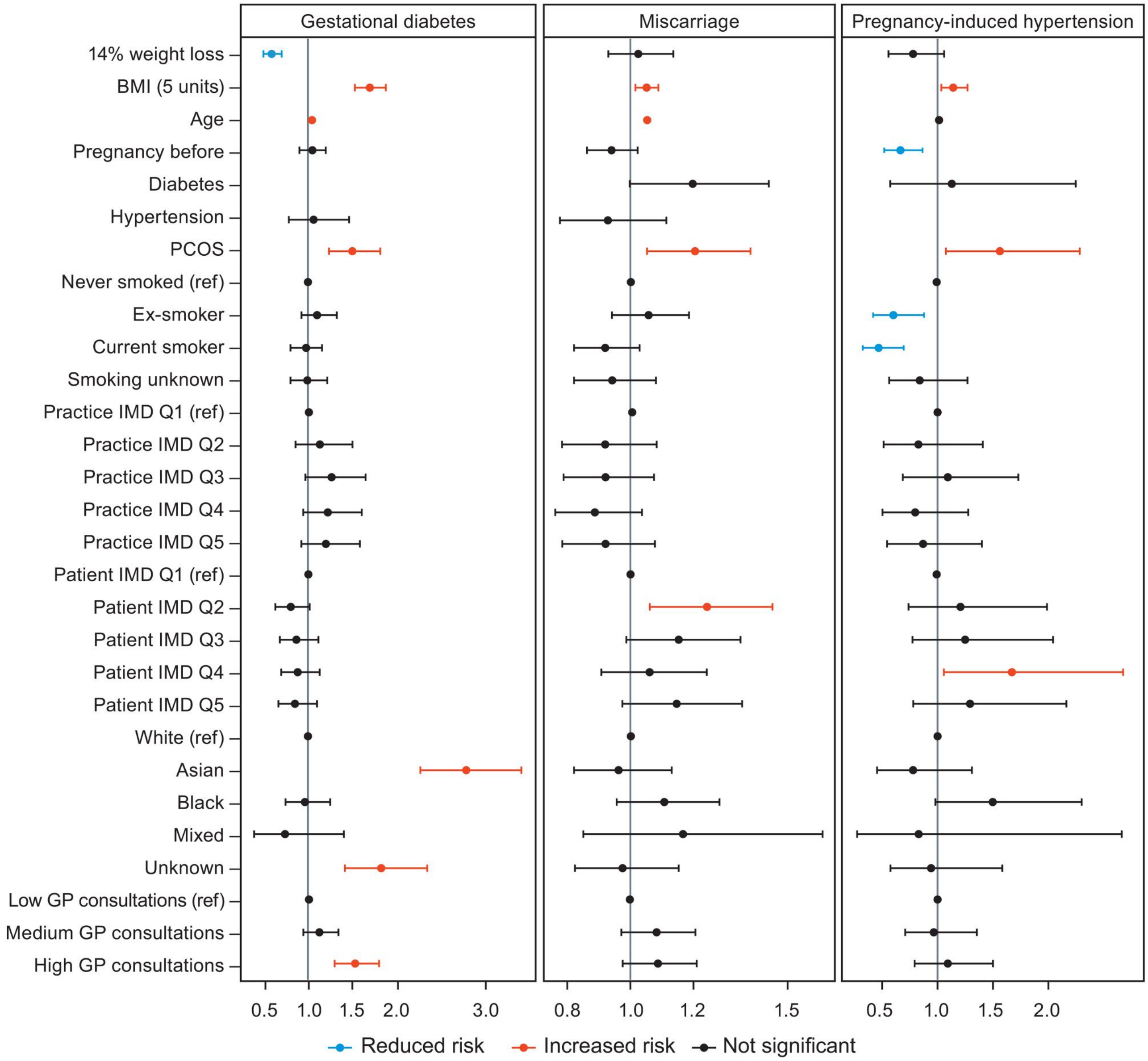


**Supplementary Figure S6. Weight loss and the risk of pregnancy complications.** GP, general practitioner; IMD, Index of Multiple Deprivation; PCOS, polycystic ovary syndrome; Q, quintile; ref, reference.
