# Supplementary material for: Association between weight loss and reproductive outcomes among women with overweight or obesity: a cohort study using UK real-world data
Source: Hum Reprod. 2025 Jul 6;40(9):1753–61. doi: 10.1093/humrep/deaf122 (PMC12408893; doi:10.1093/humrep/deaf122)
Supplement: deaf122_Supplementary_Table_S1 [file deaf122_supplementary_table_s1.pdf]

OXFORD
human reproduction

Human Reproduction, 2025, 1–1
https://doi.org/10.1093/humrep/deaf122
Supplementary Data

Supplementary Table S1. Additional demographics and baseline characteristics in the primary analysis cohort.

Variable, n (%)	Total (N = 246 670)	Stable weight[a] (N = 195 666)	Weight loss[b] (N = 51 004)
Ethnicity, n (%)			
White	195 339 (79.2)	152 831 (78.1)	42 508 (83.3)
Asian	11 795 (4.8)	9954 (5.1)	1841 (3.6)
Black	14 672 (5.9)	12 495 (6.4)	2177 (4.3)
Unknown	21 748 (8.8)	17 880 (9.1)	3868 (7.6)
Patient residence area socio-economic status			
Patient IMD 1	37 265 (15.1)	29 599 (15.1)	7666 (15.0)
Patient IMD 2	43 777 (17.7)	34 499 (17.6)	9278 (18.2)
Patient IMD 3	46 385 (18.8)	36 675 (18.7)	9710 (19.0)
Patient IMD 4	56 474 (22.9)	45 154 (23.1)	11 320 (22.2)
Patient IMD 5	57 239 (23.2)	45 442 (23.2)	11 797 (23.1)
Patient IMD unknown	5530 (2.2)	4297 (2.2)	1233 (2.4)
Practice location area socio-economic status			
Practice IMD 1	33 326 (13.5)	26 330 (13.5)	6996 (13.7)
Practice IMD 2	36 553 (14.8)	28 805 (14.7)	7748 (15.2)
Practice IMD 3	48 563 (19.7)	38 348 (19.6)	10 215 (20.0)
Practice IMD 4	60 595 (24.6)	48 271 (24.7)	12 324 (24.2)
Practice IMD 5	66 604 (27.0)	53 104 (27.1)	13 500 (26.5)
Practice IMD unknown	1029 (0.4)	808 (0.4)	221 (0.4)
Frequency of primary-care consultations during baseline period			
Low	89 428 (36.3)	72 235 (36.9)	17 193 (33.7)
Medium	77 137 (31.3)	61 143 (31.2)	15 994 (31.4)
High	80 105 (32.5)	62 288 (31.8)	17 817 (34.9)

[a] <3% weight change.
[b] 10–25% weight loss.
IMD, Index of Multiple Deprivation.
