# Supplementary material for: Association between weight loss and reproductive outcomes among women with overweight or obesity: a cohort study using UK real-world data
Source: Hum Reprod. 2025 Jul 6;40(9):1753–61. doi: 10.1093/humrep/deaf122 (PMC12408893; doi:10.1093/humrep/deaf122)
Supplement: deaf122_Supplementary_Table_S2 [file deaf122_supplementary_table_s2.pdf]

OXFORD human reproduction

Human Reproduction, 2025, 1–1
https://doi.org/10.1093/humrep/deaf122
Supplementary Data

Supplementary Table S2. Additional characteristics of women evaluated for associations with pregnancy complications (those who had a pregnancy during the 3-year follow-up period that lasted for at least 90 days).

Variable, n (%)	Total (N = 12 224)	Stable weight[a] (N = 9452)	Weight loss[b] (N = 2772)
Ethnicity, n (%)			
White	9427 (77.1)	7115 (75.3)	2312 (83.4)
Asian	989 (8.1)	824 (8.7)	165 (6.0)
Black	878 (7.2)	742 (7.9)	136 (4.9)
Unknown	745 (6.1)	619 (6.5)	126 (4.5)
Patient residence area socio-economic status			
Patient IMD 1	1822 (14.9)	1404 (14.9)	418 (15.1)
Patient IMD 2	1953 (16.0)	1488 (15.7)	465 (16.8)
Patient IMD 3	2149 (17.6)	1647 (17.4)	502 (18.1)
Patient IMD 4	2847 (23.3)	2245 (23.8)	602 (21.7)
Patient IMD 5	3211 (26.3)	2492 (26.4)	719 (25.9)
Patient IMD unknown	242 (2.0)	176 (1.9)	66 (2.4)
Practice location area socio-economic status			
Practice IMD 1	1502 (12.3)	1127 (11.9)	375 (13.5)
Practice IMD 2	1672 (13.7)	1287 (13.6)	385 (13.9)
Practice IMD 3	2354 (19.3)	1799 (19.0)	555 (20.0)
Practice IMD 4	2998 (24.5)	2354 (24.9)	644 (23.2)
Practice IMD 5	3634 (29.7)	2838 (30.0)	796 (28.7)
Practice IMD unknown	64 (0.5)	47 (0.5)	17 (0.6)
Frequency of primary-care consultations during baseline period			
Low	3990 (32.6)	3120 (33.0)	870 (31.4)
Medium	3883 (31.8)	3010 (31.8)	873 (31.5)
High	4351 (35.6)	3322 (35.1)	1029 (37.1)

[a] <3% weight change.
[b] 10–25% weight loss.
IMD, Index of Multiple Deprivation.
