# Supplementary material for: Association between weight loss and reproductive outcomes among women with overweight or obesity: a cohort study using UK real-world data
Source: Hum Reprod. 2025 Jul 6;40(9):1753–61. doi: 10.1093/humrep/deaf122 (PMC12408893; doi:10.1093/humrep/deaf122)
Supplement: deaf122_Supplementary_Table_S3 [file deaf122_supplementary_table_s3.pdf]

OXFORD

human reproduction

Human Reproduction, 2025, 1–1
https://doi.org/10.1093/humrep/deaf122
Supplementary Data

Supplementary Table S3. The contributions of all covariates and interaction terms in the Cox proportional hazard model for the primary objective.

Covariate	HR (95% CI)	P-value
**14% weight loss**	1.05 (1.02, 1.09)	0.003
**BMI (5 units)**	0.85 (0.83, 0.87)	<0.001
**Age 18–22**	0.60 (0.57, 0.64)	<0.001
**Age 23–27**	0.88 (0.84, 0.93)	<0.001
**Age 28–32 (ref)**	1.00 (1.00, 1.00)	–
**Age 33–36**	0.54 (0.51, 0.58)	<0.001
**Age 37–40**	0.22 (0.20, 0.24)	<0.001
**Pregnancy before**	1.19 (1.13, 1.25)	<0.001
**Diabetes**	0.95 (0.89, 1.01)	ns
**Hypertension**	0.86 (0.81, 0.92)	<0.001
**PCOS**	1.15 (1.10, 1.21)	<0.001
**Never smoked (ref)**	1.00 (1.00, 1.00)	–
**Ex-smoker**	1.04 (1.00, 1.08)	ns
**Current smoker**	0.95 (0.92, 0.99)	0.005
**Smoking unknown**	0.92 (0.89, 0.97)	<0.001
**Practice IMD Q1 (ref)**	1.00 (1.00, 1.00)	–
**Practice IMD Q2**	1.01 (0.96, 1.07)	ns
**Practice IMD Q3**	1.00 (0.95, 1.05)	ns
**Practice IMD Q4**	1.00 (0.95, 1.05)	ns
**Practice IMD Q5**	0.97 (0.92, 1.02)	ns
**Practice IMD unknown**	1.32 (1.06, 1.64)	0.01
**Patient IMD Q1 (ref)**	1.00 (1.00, 1.00)	–
**Patient IMD Q2**	0.92 (0.87, 0.96)	<0.001
**Patient IMD Q3**	0.90 (0.86, 0.95)	<0.001
**Patient IMD Q4**	0.91 (0.87, 0.96)	<0.001
**Patient IMD Q5**	0.91 (0.86, 0.96)	<0.001
**Patient IMD unknown**	0.90 (0.81, 1.00)	ns
**White (ref)**	1.00 (1.00, 1.00)	–
**Asian**	1.19 (1.14, 1.26)	<0.001
**Black**	1.21 (1.15, 1.27)	<0.001
**Mixed**	1.05 (0.95, 1.17)	ns
**Ethnicity unknown**	0.75 (0.71, 0.79)	<0.001
**Low GP consultations (ref)**	1.00 (1.00, 1.00)	–
**Medium GP consultations**	1.06 (1.03, 1.10)	<0.001
**High GP consultations**	1.13 (1.09, 1.17)	<0.001
**14% weight loss: BMI**	1.01 (1.00, 1.02)	<0.001
**BMI: Age 18–22**	1.03 (1.02, 1.04)	<0.001
**BMI: Age 23–27**	1.01 (1.01, 1.02)	<0.001
**BMI: Age 28–32 (ref)**	1.00 (1.00, 1.00)	–
**BMI: Age 33–36**	1.00 (1.00, 1.01)	ns
**BMI: Age 37–40**	1.02 (1.01, 1.02)	<0.001
**Age 18–22: Pregnancy before**	2.66 (2.44, 2.89)	<0.001
**Age 23–27: Pregnancy before**	1.69 (1.57, 1.81)	<0.001
**Age 28–32: Pregnancy before (ref)**	1.00 (1.00, 1.00)	–
**Age 33–36: Pregnancy before**	0.94 (0.86, 1.02)	ns
**Age 37–40: Pregnancy before**	0.96 (0.86, 1.07)	ns

GP, general practitioner; HR, hazard ratio; IMD, Index of Multiple Deprivation; ns, not significant; PCOS, polycystic ovary syndrome; Q, quintile; ref, reference.
