# Supplementary material for: Association between weight loss and reproductive outcomes among women with overweight or obesity: a cohort study using UK real-world data
Source: Hum Reprod. 2025 Jul 6;40(9):1753–61. doi: 10.1093/humrep/deaf122 (PMC12408893; doi:10.1093/humrep/deaf122)
Supplement: deaf122_Supplementary_Table_S4 [file deaf122_supplementary_table_s4.pdf]

OXFORD
human reproduction

Human Reproduction, 2025, 1–1
https://doi.org/10.1093/humrep/deaf122
Supplementary Data

Supplementary Table S4. The contributions of all covariates in the logistic regression models for estimating the association with the risk of pregnancy complications.

	Gestational diabetes		Miscarriage		Pregnancy-induced hypertension	
	OR (95% CI)	P-value	OR (95% CI)	P-value	OR (95% CI)	P-value
14% weight loss	0.58 (0.48, 0.70)	<0.001	1.03 (0.93, 1.14)	ns	0.77 (0.55, 1.07)	ns
Increase in baseline BMI (5 units)	1.69 (1.52, 1.88)	<0.001	1.05 (1.01, 1.09)	0.008	1.14 (1.03, 1.26)	0.01
Age	1.03 (1.02, 1.05)	<0.001	1.05 (1.05, 1.06)	<0.001	1.00 (0.98, 1.03)	ns
Pregnancy before	1.04 (0.90, 1.20)	ns	0.94 (0.86, 1.02)	ns	0.67 (0.51, 0.87)	0.003
Diabetes	–	–	1.20 (0.99, 1.44)	ns	1.13 (0.56, 2.26)	ns
Hypertension	1.06 (0.76, 1.46)	ns	0.93 (0.77, 1.12)	ns	–	–
PCOS	1.50 (1.23, 1.83)	<0.001	1.20 (1.05, 1.38)	0.009	1.56 (1.06, 2.29)	0.02
Never smoked (ref)	1.00 (1.00, 1.00)	–	1.00 (1.00, 1.00)	–	1.00 (1.00, 1.00)	–
Ex-smoker	1.10 (0.91, 1.33)	ns	1.06 (0.94, 1.19)	ns	0.61 (0.41, 0.89)	0.01
Current smoker	0.97 (0.80, 1.17)	ns	0.92 (0.82, 1.03)	ns	0.47 (0.32, 0.70)	<0.001
Smoking unknown	0.98 (0.79, 1.22)	ns	0.94 (0.82, 1.08)	ns	0.84 (0.56, 1.27)	ns
Practice IMD Q1 (ref)	1.00 (1.00, 1.00)	–	1.00 (1.00, 1.00)	–	1.00 (1.00, 1.00)	–
Practice IMD Q2	1.12 (0.84, 1.50)	ns	0.92 (0.78, 1.08)	ns	0.83 (0.49, 1.40)	ns
Practice IMD Q3	1.26 (0.96, 1.65)	ns	0.92 (0.78, 1.08)	ns	1.09 (0.68, 1.73)	ns
Practice IMD Q4	1.23 (0.94, 1.60)	ns	0.89 (0.76, 1.04)	ns	0.79 (0.49, 1.28)	ns
Practice IMD Q5	1.20 (0.91, 1.58)	ns	0.92 (0.78, 1.08)	ns	0.86 (0.53, 1.41)	ns
Practice IMD unknown	2.41 (0.71, 8.23)	ns	1.16 (0.60, 2.25)	ns	2.64 (0.54, 12.87)	ns
Patient IMD Q1 (ref)	1.00 (1.00, 1.00)	–	1.00 (1.00, 1.00)	–	1.00 (1.00, 1.00)	–
Patient IMD Q2	0.80 (0.61, 1.03)	ns	1.24 (1.06, 1.45)	0.008	1.21 (0.73, 2.00)	ns
Patient IMD Q3	0.87 (0.68, 1.12)	ns	1.15 (0.98, 1.35)	ns	1.26 (0.76, 2.06)	ns
Patient IMD Q4	0.88 (0.69, 1.13)	ns	1.06 (0.90, 1.25)	ns	1.67 (1.04, 2.69)	0.03
Patient IMD Q5	0.85 (0.66, 1.10)	ns	1.15 (0.97, 1.35)	ns	1.30 (0.77, 2.17)	ns
Patient IMD unknown	0.38 (0.17, 0.85)	0.02	1.14 (0.80, 1.61)	ns	1.40 (0.47, 4.13)	ns
White (ref)	1.00 (1.00, 1.00)	–	1.00 (1.00, 1.00)	–	1.00 (1.00, 1.00)	–
Asian	2.78 (2.25, 3.42)	<0.001	0.96 (0.82, 1.13)	ns	0.78 (0.47, 1.31)	ns
Black	0.96 (0.73, 1.27)	ns	1.10 (0.95, 1.28)	ns	1.50 (0.97, 2.31)	ns
Mixed	0.73 (0.38, 1.41)	ns	1.17 (0.84, 1.61)	ns	0.84 (0.26, 2.66)	ns
Ethnicity unknown	1.82 (1.41, 2.35)	<0.001	0.98 (0.82, 1.16)	ns	0.95 (0.57, 1.59)	ns
Low GP consultations (ref)	1.00 (1.00, 1.00)	–	1.00 (1.00, 1.00)	–	1.00 (1.00, 1.00)	–
Medium GP consultations	1.12 (0.93, 1.35)	ns	1.08 (0.97, 1.21)	ns	0.98 (0.71, 1.36)	ns
High GP consultations	1.52 (1.28, 1.81)	<0.001	1.09 (0.98, 1.21)	ns	1.10 (0.80, 1.52)	ns

GP, general practitioner; IMD, Index of Multiple Deprivation; ns, not significant; OR, odds ratio; PCOS, polycystic ovary syndrome; Q, quintile; ref, reference.
